# P-1844. Performance Characteristics of a Validated Pan-Genotypic Real-Time RT-PCR Assay for the Quantification of Hepatitis Delta Virus from Human Serum and Plasma

**DOI:** 10.1093/ofid/ofaf695.2013

**Published:** 2026-01-11

**Authors:** Lydia Sweet, Arsalan Khan, Gerald Wallweber, Christos J Petropoulos

**Affiliations:** Labcorp-Monogram Biosciences, South San Francisco, CA; Labcorp, South San Francisco, California; Labcorp - Monogram Biosciences, South San Francisco, California; Labcoorp-Monogram Biosciences, South San Francisco, California

## Abstract

**Background:**

Hepatitis Delta Virus (HDV) is a 1.7 kb single-stranded, circular RNA virus that relies on Hepatitis B Virus (HBV) surface antigens (HBsAg) for encapsulation, and therefore is dependent on co-infection with HBV for replication and transmission. Globally, it is estimated that 12-60 million individuals are infected with HDV. There are eight HDV genotypes with distinct and overlapping geographical distributions. Bulevirtide (Hepcludex) is the only approved treatment for HDV infection and currently only in Europe. Testing for HDV is expected to increase once bulevirtide and additional therapies are available. We describe the performance characteristics of a new laboratory-developed test for the quantification of HDV RNA

**Methods:**

Total nucleic acid is isolated from human serum and EDTA-plasma with an improved semi-automated extraction protocol on the KingFisher Flex. Samples are spiked with bacteriophage MS2 to monitor performance of the entire procedure. HDV and MS2 RNA are detected using a 2-step RT-PCR assay optimized for the QuantStudio 7 Flex Real-Time PCR System and results report in IU/mL. Assay performance has been validated for accuracy, precision, linearity, sensitivity, specificity and genotype inclusivity.

**Results:**

Matched sample types resulted in similar viral loads; average bias 0.04 log_10_ IU/mL. The LOD was 8.81 and 9.50 IU/mL for serum and plasma respectively, with an ULOQ of > 2x10^6^ IU/mL for both. Results from a comparator laboratory were similar, slope 1.103 log_10_ IU/mL and average bias 0.6 log_10_ IU/mL Results from spike-recovery, precision, cross-reactivity and interfering substances were also similar between sample types. Transcripts from 22 HDV reference plasmids representing all HDV genotypes and subtypes were linear over a 5 – 7 log_10_ range, R squared > 0.99. PCR efficiencies from 21/22 transcripts were > 90%, genotype 4b had a PCR efficiency of 86%.

**Conclusion:**

The quantitative HDV RNA assay demonstrated acceptable analytical performance as well as inclusivity for all HDV genotypes. The assay has been validated to CLIA/CAP specifications and is suitable for quantification of HDV RNA from human serum and EDTA-plasma.

**Disclosures:**

Lydia Sweet, PhD, Labcorp-Monogram Biosciences: Employee Arsalan Khan, BSc, Labcorp-Monogram Biosciences: Employee Gerald Wallweber, PhD, Labcorp-Monogram Biosciences: employee Christos J. Petropoulos, Ph.D., Labcorp-Monogram Biosciences: employee

